# Genetically encoded mediators for sonogenetics and their applications in neuromodulation

**DOI:** 10.3389/fncel.2023.1326279

**Published:** 2023-12-22

**Authors:** Hsien-Chu Wang, Thi-Nhan Phan, Chi-Ling Kao, Chih-Kuang Yeh, Yu-Chun Lin

**Affiliations:** ^1^Institute of Molecular Medicine, National Tsing Hua University, Hsinchu, Taiwan; ^2^Department of Biomedical Engineering and Environmental Sciences, National Tsing Hua University, Hsinchu, Taiwan; ^3^Department of Medical Science, National Tsing Hua University, Hsinchu, Taiwan

**Keywords:** ultrasound, sonogenetics, gas vesicles, prestin, mechanosensitive ion channels

## Abstract

Sonogenetics is an emerging approach that harnesses ultrasound for the manipulation of genetically modified cells. The great penetrability of ultrasound waves enables the non-invasive application of external stimuli to deep tissues, particularly advantageous for brain stimulation. Genetically encoded ultrasound mediators, a set of proteins that respond to ultrasound-induced bio-effects, play a critical role in determining the effectiveness and applications of sonogenetics. In this context, we will provide an overview of these ultrasound-responsive mediators, delve into the molecular mechanisms governing their response to ultrasound stimulation, and summarize their applications in neuromodulation.

## Introduction

The development of techniques for manipulating the activities of target neurons is crucial for understanding neuronal circuits in the brain and offering potential therapeutic applications for brain-related disorders. Optogenetics is a well-established approach that employs light to control target cells with artificially expressed photosensitive proteins (Pastrana, 2011). It has provided precise control over neuronal cells *in vitro* and *in vivo*, advancing the field of neuroscience and offering novel strategies for treating various diseases (Mazzitelli et al., 2022; Mirzayi et al., 2022; Kim et al., 2023). However, this powerful tool faces limitations due to the poor penetration of light, which restricts its applications in deep tissues. Significant efforts have been dedicated to achieving non-invasive neuromodulation. For example, thermogenetics, magnetogenetics, and sonogenetics combine various external stimuli with genetic techniques to non-invasively stimulate cells buried in deep tissues (Bernstein et al., 2012; Azadeh et al., 2021; Del Sol-Fernández et al., 2022). Among these stimuli, ultrasound (US) has received approval from the Food and Drug Administration for clinical applications in various human diseases, including Parkinson's diseases, dyskinesia, essential tremor, and tremor-dominant Parkinson's disease. Furthermore, the US has exhibited significant potential in the control of pain (Petterson et al., 2020), histotripsy (Vidal-Jove et al., 2022), and thermoablation (Jung et al., 2015). Due to its substantial clinical promise, sonogenetics has garnered growing attention. This review will focus on sonogenetics and provide an overview of genetically encoded mediators that sensitize cells to ultrasound (US) stimulation.

## Ultrasound and sonogenetics

**Figure 1:**
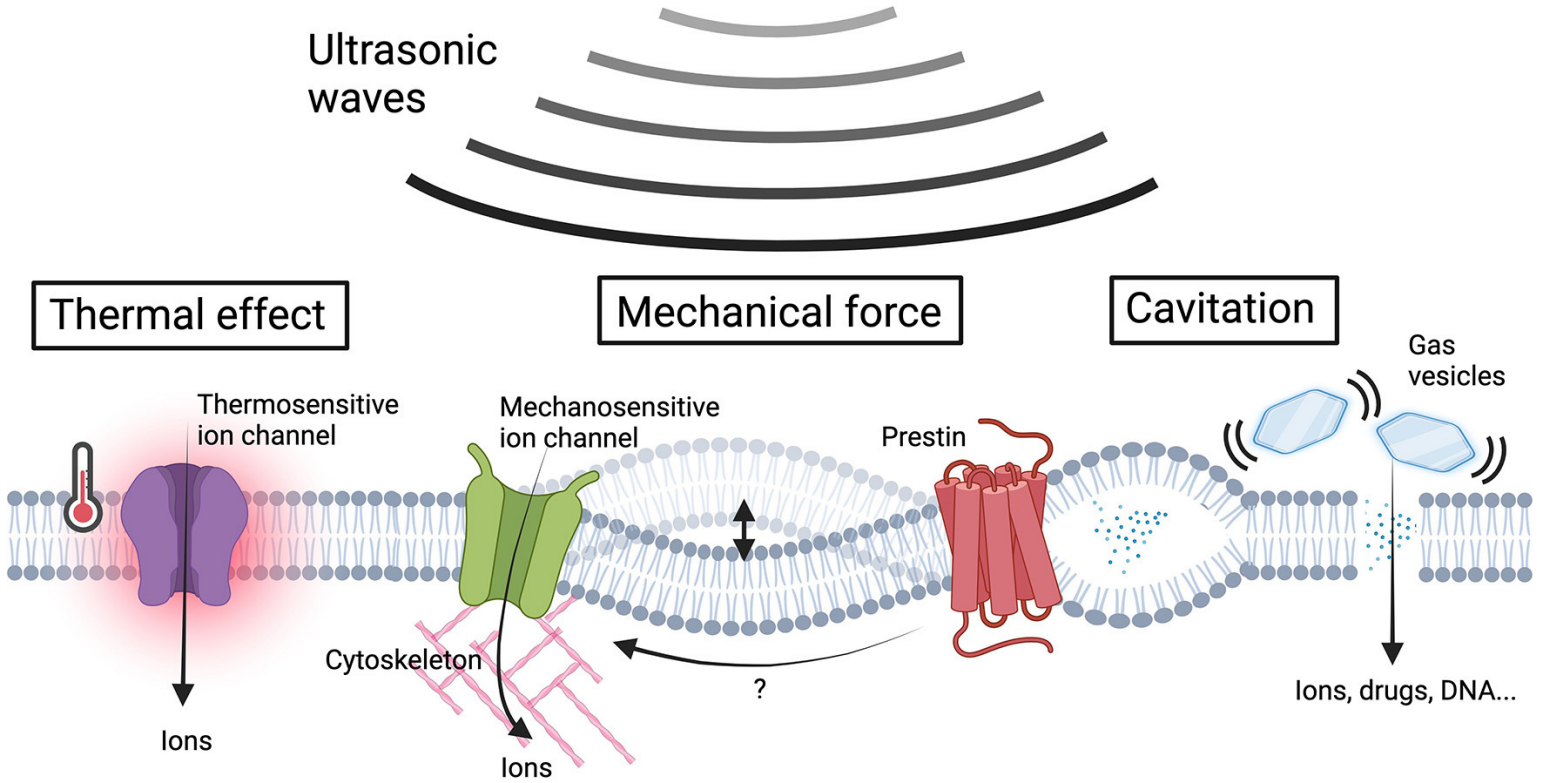
US-induced bio-effects and sensing proteins in sonogenetics. Ultrasonic waves give rise to a variety of bio-effects including thermal effects, membrane cavitation, and the generation of mechanical forces to cellular components. The US-induced heating activates thermosensitive ion channels, resulting in ion influx. The US energy could covert to mechanical forces that induce membrane deflection or intramembrane cavitation and subsequently activate mechanosensitive ion channels to facilitate ion influx. The increase in membrane tension induces transmembrane voltage fluctuations, which can be sensed by prestin, triggering downstream calcium influx through its electromobility. Gas vesicles vibrate in response to acoustic pressures, improving membrane permeability for the influx of ions and membrane impermeable molecules. The US-induced mechanical force effects can be further intensified by gas vesicles, promoting ion influx through mechanosensitive ion channels. The ion influx resulting from the aforementioned mechanisms alters the membrane potential, ultimately leading to neuromodulation (Figure created by BioRender.com).

US are acoustic waves with frequencies above the upper limit of human hearing. US waves efficiently propagate several centimeters deep at a speed of approximately 1.5 km/sec within soft tissues. An annular array of multiple transducers can deliver US waves to defined small tissue volumes and is therefore referred to as focused US (FUS). Due to its exceptional penetrability and spatiotemporal resolution, FUS has been widely employed in diagnostic imaging. The penetrability and spatial resolution of FUS are determined by its frequency. Higher frequency US provides better resolution but limited penetrability, while lower frequency US offers relatively poor resolution but excellent penetrability (Boissenot et al., 2016). This trade-off explains why high-frequency and low-frequency FUS are typically used for superficial and deep tissue imaging, respectively. In addition to imaging, accumulating reports have shown that low-intensity FUS (averaging < 100 W/cm^2^ acoustic pressure over the pulse train) can induce a wide range of bio-effects, including heating, mechanical forces, and cavitation (Figure 1) (Collins and Mesce, 2022). While the thermal effect is known to be triggered by continuous FUS at high frequencies (Pinton et al., 2010; Rossmanna and Haemmerich, 2014), the parameters of FUS that specifically cause other bio-effects are not fully understood (Chu et al., 2022). This unpredictability may result from variations in acoustic properties or the expression profiles of endogenous US-sensing proteins in different tissues. Consequently, opposite effects on neuromodulation in distinct tissues can be triggered by similar US stimulation parameters. For example, low-intensity US (<17 W/cm^2^) has been found to produce contrasting neuromodulation effects in different brain regions (details summarized in Wang et al., 2020). One strategy to address this challenge is the overexpression of heterogeneous US-sensing proteins that are expected to be responsive to US-induced bio-effects in target cells, thereby ensuring the desired neuromodulation via FUS stimulation. Furthermore, genetic tools enable the precise expression of US-sensing proteins in specific cell types, providing target cells with enhanced US sensitivity compared to naïve cells. This opens up the possibility of using FUS to intendedly activate genetically modified target cells but not naïve cells within FUS-illuminated regions. This review will provide an overview of several genetically encoded US-sensing proteins that are responsive to different US-indued bio-effects and describe their applications in neuromodulation.

## Thermal effect-based sonogenetics

High-frequency US encounters significant absorption and scattering in tissues during propagation, resulting in the conversion of acoustic energy into heat. High-intensity FUS can rapidly elevate temperatures to the range of 55~80°C within the focal zone, leading to deleterious thermal effects for ablating diseased tissues (Bystritsky and Korb, 2015). By carefully tuning the US parameters, low-intensity FUS can induce localized noxious heat at approximately 42°C. Neuronal cells detect such temperature elevation through thermosensitive transient receptor potential (TRP) cationic channels (Zhu et al., 2019). Among the TRP family members, TRPV1 (Transient Receptor Potential Vanilloid 1) is a nonselective ligand-gated cation channel highly expressed in peripheral neurons and various brain regions (Fernandes et al., 2012; Pecze et al., 2016; Roet et al., 2019). TRPV1 can be activated by warm stimuli (~42°C) and regulate neuronal synaptic activities. Through viral overexpression of exogenous TRPV1 in the mouse cortex, several studies achieved successful activation of target neurons with genetic modification and modulation of animal locomotor behavior via FUS-induced thermal effects (Yang et al., 2021; Xu et al., 2023). It is noteworthy that optimized FUS stimulation (0.7 MPa) is necessary for specific modulation in genetically defined neurons, as naive mice are responsive to stronger FUS stimulation (1.1 MPa), possibly due to the contribution of endogenous thermosensors (Xu et al., 2023). The safety of this thermal modulation treatment can be secured by carefully tuning FUS parameters (Yang et al., 2021; Xu et al., 2023).

## Mechanical force-based sonogenetics

Acoustic waves generate physical momentum when they encounter obstacles along their path, thus converting sound waves into mechanical forces that affect the given tissues. These obstacles can be attributed to biomolecules or materials with acoustic properties. The effects of mechanical forces on cells and tissues have been extensively studied, as they influence various biocomponents such as cell membranes (Vasan et al., 2022), cytoskeletons (Clark et al., 2008; Liang et al., 2013; Chuang and Chen, 2022), and the extracellular matrix (Chuang and Chen, 2022), subsequently altering the properties of connecting mechanosensors. The identification of numerous mechanosensitive ion channels has provided insights into the molecular mechanisms of cell mechanosensing (Lim et al., 2018). The recent research using high-speed imaging has provided direct evidence that US induces cell membrane deflection, resulting in membrane depolarization and neuron excitation (Vasan et al., 2022). Furthermore, Sorum et al. demonstrated that US activates a mechanosensitive channel known as TRAAK (TWIK-related arachidonic acid-activated K^+^ channel) (Sorum et al., 2021), and this activation depends on membrane structures. These studies support the hypothesis that US mechanically affects cell components, leading to the activation of mechanosensitive ion channels and downstream signaling pathways. Based on this working mechanism, numerous studies have identified several mechanosensitive ion channels that are involved in US sensing (Table 1).

**Table 1:** Summary of genetically encoded US sensing proteins and their applications in neuromodulation.

**US-induced bio-effects**	**US-sensing proteins**	**Ultrasound parameters**			**Applications in neuromodulation**	**References**
		**Frequency**	**Duration**	**Pressure and others**		
Thermal effect	TRPV1	1.5 and 1.7 MHz	30 s	>0.9 MPa PRF 10 Hz 40% duty cycle	• US induced calcium influx in mouse cortex. • US reversibly and repeatedly evoked rational behavior in TRPV1^+^ mice.	Yang et al., 2021
Mechanical force	MscL-I92L	29.92 MHz	50, 100, 200, 300, 400 ms	0.12–0.45 MPa PRF 1, 5, and 10 Hz Surface acoustic wave	• US evoked the spikes in rat primary hippocampal neurons.	Ye et al., 2018
	MscL-G22S	0.5, 2.25 and 15 MHz	10 s	>0.1 MPa PRF 1 kHz 50% duty cycle	• US stimulation of the primary rat retina neurons *ex vivo* and visual cortex of rodents *in vivo* generated light perception for vision restoration by electrophysiological and muti-electrode array recording. • US increased water reward behavior in G22S MscL-transfected mice.	Cadoni et al., 2023
		0.5 MHz	300 ms	0.025–0.5 MPa PRF 1 kHz 40% duty cycle	• US induced calcium influx in primary cortical neurons expressing MscL-G22S. • US increased c-fos level in neuron cells expressing MscL-G22S in the mouse dorsomedial straitum. • US stimulated excitatory neurons in M1 mouse brain region expressing MscL-G22S, resulting in significant muscular responses in mouse limb.	Qiu et al., 2020
		0.5 and 0.9 MHz	300 ms	0.05–0.35 MPa PRF 1 kHz 40 or 50% duty cycle	• US induced calcium influx in subcortical neurons expressing MscL-G22S. • US manipulated neural activity and increased c-fos signal in dorsal striatum and subthalamic nuclei brain region to improve motor coordination and mobility of Parkinson's mouse model.	Xian et al., 2023
	Piezo1	0.5 MHz	200 ms	0.1–0.5 MPa PRF 1 kHz 40% duty cycle	• US induced calcium influx in mHippoE-18 cells (an embryonic mouse hippocampal cell line) and primary cortical neurons expressing Piezo1.	Qiu et al., 2019
		0.5 MHz	50, 200, 500 ms	0.2–0.8 MPa PRF 1 kHz 50% duty cycle	• Genetic depletion of piezo1 reduced the US responses, including neuronal calcium responses, limb movement, and muscle electromyogram (EMG) responses.	Zhu et al., 2023
	TRP-4	0.69–3 MHz	10 ms	0–0.9 MPa Continuous wave	• US activated PVD sensory neurons and AIY neurons expressing TRP-4 to induce reversal behavior of MB-bound *C. elegans*.	Ibsen et al., 2015
	MEC-4 and TRP-4	10 MHz	200 ms	0.2–1.0 MPa 1 kHz 50% duty cycle	• US activated MEC-4 in touch receptor neurons and induced reversal behavior of *C. elegans*.	Kubanek et al., 2018
	MEC-4 and TRP-4	2.25 and 10 MHz	10 ms	0.79–1.0 MPa	• US triggered MEC-4 and TRP-4 dependent reversal response of *C. elegans*.	Magaram et al., 2022
	MEC-4	27.4 MHz	6.4 ms	Surface acoustic wave	• US activated and increased calcium influx of ALM and PLM neurons expressing MEC-4 and MEC-6 mutants.	Zhou et al., 2022
	K^+^ mechanosensitive ion channels	1 MHz	10 min	50 mW/cm^2^ PRF 100 Hz 20% duty cycle	• Low-intensity pulsed US inhibited the neurotoxicity and mitochondrial dysfunction caused by a dopaminergic neuronal toxin, 1-Methyl-4-phenylpyridinium in PC12 cells.	Zhao et al., 2017
		43 MHz	1 s	50 W/cm^2^	• US increased action potential firing rate in CA1 pyramidal neurons. • US activated thermo- and mechano-sensitive K2P channels on brain tissue.	Prieto et al., 2020
	ASIC1a	1 MHz	3 s	12 kPa PRF 1 kHz 20% duty cycle I_SPPA_ = 7.4 mW/cm^2^	• US stimulation increased p-ERK signal in mouse cortex, hippocampus, and amygdala. • Low-intensity US activated mechanotransduction and cultured neurons through ASIC1a and cytoskeletal proteins.	Lim et al., 2021
	TRAAK	5 MHz	10 ms	0.2–3.6 W/cm^2^ Continuous wave	• US can activate the K^+^ channel TRAAK in mouse cortical-layer 2/3 pyramidal neurons.	Sorum et al., 2021
	Mouse TRPA1	0.35, 0.43, and 0.5 MHz	100 ms	>0.01 MPa PRF 1.16, 1.5 and 2 kHz 50% duty cycle	• Low-intensity, low-frequency US affects brain activity by opening TRPA1 channels in astrocytes, resulting in glutamate releasing for neuromodulation and tail movement.	Oh et al., 2019
	Human TRPA1	1, 2, and 7 MHz	100 ms	0.15–2.5 MPa Continuous wave	• US induced calcium influx in mouse primary cortical neurons. • US induced limb responses in mice with hsTRPA1 expression.	Duque et al., 2022
	Voltage-gated Na^+^ channels	0.44-0.67 MHz	5 s	< 1 MPa PRF 0–100 Hz	• US increased Na^+^ transients in primary hippocampal CA1 pyramidal neurons and in brain slices.	Tyler et al., 2008
	TRPV1, TRPV2, TRPV4, TRPC1, Piezo1, TRPM7 and TRPP1/2 complex	0.3 and 0.67 MHz	500 ms	0–15 W/cm^2^ continuous wave PRF 1 and 1.5 kHz	• US increase calcium influx through the channels like TRPP1/2, TRPC1, and Piezo1 in primary cultured cortical neurons. • Activate mouse primary cortical neurons by overexpressing TRPC1, TRPP2, and TRPM4 in primary cultured cortical neurons reduce the threshold of US activation.	Yoo et al., 2022
	Microbubbles and Piezo1	2 MHz	10 s	0.03, 0.06, 0.11, and 0.17 MPa PRF 10 Hz 5% duty cycle	• US stimulation at lower intensity triggered calcium response in Neuron-2A cells and primary hippocampal neurons bound with Piezo1-targeted microbubbles.	Shen et al., 2021
	Gas vesicles and MscL-G22S	1 MHz	3 s	0.07–0.28 MPa PRF 1 kHz 10% duty cycle	• US induced calcium influx in mHippoE-18 cells (an embryonic mouse hippocampal cell line) and rat primary cortical neurons. • US increased c-fos level in primary neurons in the presence of gas vesicles and MscL-G22S. • US activated neurons in ventral tegmental area of mouse brains in the presence of gas vesicles and MscL-G22S.	Hou et al., 2021
Electromotility	Prestin (N7T, N308S)	0.5 MHz	3 s	0.5 MPa PRF 10 Hz 10% duty cycle	• US induced membrane potential changes in SHSY5Y cells. • US increased c-fos level in ventral tegmental area of mouse brains. • US activated dopaminergic neurons in substantia nigra and ameliorated the dopaminergic neurodegeneration in Parkinson's disease mice. • US mitigated the motor symptoms of Parkinson's disease mice.	Huang et al., 2020, 2021; Wu et al., 2020; Fan et al., 2021

US-sensitive mechanosensors have also shown their value in neuromodulation. The expression of TRP-4 in sensory neurons of C. elegans allows them to respond to US stimulation, resulting in reversed motor behavior in nematodes (Ibsen et al., 2015; Magaram et al., 2022). Yoo et al. confirmed that the overexpression of TRPC1, TRPP2, and TRPM4 in primary cultured cortical neurons makes them sensitive to US stimulation (Yoo et al., 2022). Oh et al. demonstrated that low-intensity and low-frequency US activates endogenous TRPA1 in mouse astrocytes, leading to the release of glutamate that activates NMDA receptors in neighboring neurons for neuromodulation (Oh et al., 2019). In a separate study, Duque et al. screened 191 TRP ion channels and found that human TRPA1 outperforms other candidates in response to pulsed high-frequency and high-intensity US (100 ms duration, 7 MHz, 2.5 MPa). Ectopically expressing human TRPA1 in mouse layer V motor cortical neurons allows them to respond to transcranial US stimulation, inducing limb responses in mice (Duque et al., 2022). The significant roles of endogenous Piezo1 in US sensing have been demonstrated through genetic depletion assays in mice (Li et al., 2019; Zhu et al., 2023). However, it remains unclear whether specific neuromodulation can be triggered in Piezo1-overexpressing neurons *in vivo* due to challenges associated with viral delivery of the Piezo1 gene. Ye et al. and Qiu et al. found that MscL-I92L and MscL-G22S, two MscL variants, exhibit greater mechanosensitivity compared to the wild-type, conferring US sensitivity to genetically modified neurons (Ye et al., 2018; Qiu et al., 2020). MscL-G22S-based sonogenetics enables specific activation in the dorsal striatum and inducible mouse locomotion (Xian et al., 2023). Stimulation of MscL-G22S-transfected neurons in the subthalamus by US successfully alleviates movement symptoms in parkinsonian mice (Xian et al., 2023). The majority of these studies activate mechanosensitive ion channels with US at frequencies lower than 3 MHz and peak pressures <1 MPa, suggesting that low-intensity and low-frequency US is sufficient for activating mechanosensitive ion channels. However, the activation of TRPA1 (Duque et al., 2022), TRAAK (Sorum et al., 2021), and Piezo1 (Prieto et al., 2018; Liao et al., 2021) with high-intensity (>1 MPa) and high-frequency (4.78~43 MHz) US stimulation still offers the flexibility of neuromodulation with better spatial resolution in superficial tissues. For example, MscL-G22S has been used to activate retinal and cortical neurons (Cadoni et al., 2023). By optimizing the parameters of high-frequency US (15 MHz), the study achieves excellent spatiotemporal resolution for proper visual restoration in superficial sites (Cadoni et al., 2023). In mechanical force-based sonogenetics, many efforts have been made to reduce unintended effects in naïve cells. Microbubbles (MBs, more details in next section) have been used in conjunction with mechanosensitive ion channels to robustly activate modified cells with low-intensity US, significantly reducing non-specific effects in naive cells (Heureaux et al., 2014; Ibsen et al., 2015; Huang et al., 2021). By using heterogeneously expressing mechanosensing proteins, sonogenetics can be employed to non-invasively modulate specific neurons, target circuits, and downstream behaviors, showing promise in the treatment of brain-related diseases.

## Cavitation-based sonogenetics

MBs are particles with diameters ranging from 1 to 10 micrometers, enclosed within shells made of lipids, proteins, or polymers, and containing gaseous cores. The impedance mismatch between the gaseous core of MBs and the surrounding biological tissues causes MBs to expand and contract in response to the positive and negative phases of US waves (Wu and Nyborg, 2008; Kooiman et al., 2014). At low acoustic pressures, MBs undergo symmetrical linear oscillations, and their oscillation amplitudes are influenced by the driving acoustic pressures (Lentacker et al., 2014). As US acoustic pressure increases, the oscillations become asymmetric, promoting the expansion phase (Sboros, 2008; Lentacker et al., 2014; Gu et al., 2016), which can disturb membrane integrity (Qin et al., 2018). Additionally, the fluid micro-streaming generated by oscillating MBs can lead to membrane pore formation (Chen et al., 2016). The shear stress from microstreaming can enhance cell membrane permeability, rearrange the cytoskeleton, and promote nuclear contraction (Chen et al., 2022). Under high US acoustic pressure (several hundred kilopascals), MBs undergo asymmetric oscillations with large amplitudes, resulting in collapse and fragmentation, a phenomenon called inertial cavitation. When inertial cavitation occurs near the cell membrane, the pressure can temporarily disrupt the membrane and rupture the cytoskeleton (Fan et al., 2013). Changes in cell membrane permeability induced by US sonication with MBs can enhance ion influx, leading to membrane potential changes for neuromodulation. Shear-force-induced membrane tension variations can also activate mechanosensitive ion channels and their downstream effects.

Recently, gas vesicles (GVs) are the gas-filled protein nanostructures which naturally exist in many bacteria and archaea (Hill and Salmond, 2020) have emerged their values in US therapy. GVs have nanoscale dimensions, typically ranging from 45 to 250 nanometers in width and 100 to 600 nanometers in length (Pfeifer, 2012), and they possess a hollow structure filled with air. The shells of GVs primarily consist of hydrophobic proteins on the inner surface and hydrophilic proteins on the outer surface, rendering them highly physically stable since gases can permeate but not liquid water (Pfeifer, 2012; Shapiro et al., 2014). GVs serve as flotation devices for microorganisms in aqueous environments. The acoustic properties of GVs are remarkably similar to those of MBs, as they also have a gaseous core surrounded by liquid. As expected, it has demonstrated that US can induce cavitation effects via GVs (Hou et al., 2021). GVs produce strong ultrasonic contrast signals and have been employed as markers for various genes, chemicals, and cellular processes (Rabut et al., 2020; Wang et al., 2022). Furthermore, the oscillations of GVs have the potential to cause mechanical disturbances in their surroundings (Rabut et al., 2020). Due to these unique acoustic characteristics, GVs may amplify low-frequency US sonication, inducing oscillations and activating mechanosensitive ion channels to produce neuromodulation effects. With their nanoscale dimensions and gene-encodable structures, engineered genes can be delivered to extravascular vessels in the target area (Lakshmanan et al., 2016; Lu et al., 2018), enhancing the efficiency of targeted therapy. Additionally, GVs' shells can be chemically modified to load antibodies and biomolecule medications, making them a potentially powerful tool for neuromodulation therapeutic strategies for neural disorders. For example, a recent study combined low-intensity US sonication (1 MHz, burst width 200 μs, burst interval 2 ms, 0.28 MPa peak negative pressure) with GVs to enhance calcium influx and activate neuron activities (Hou et al., 2021). Mechanosensitive ion channels, especially the mechanosensitive MscL-G22S channel, played a critical role in inducing the stimulation effect in neurons (Hou et al., 2021). Biogenic GVs offer several advantages over MBs, as they can be precisely introduced to target tissues using genetic tools, whereas delivering MBs to extravascular tissues is challenging. Moreover, GVs exhibit better tissue penetrability due to their smaller size compared to MBs.

## Electromobility-based sonogenetics

Many US sensing proteins were explored mainly dependent on their expected responses to US-indued bio-effects. However, few studies have adopted alternative strategies to address this matter. It is well-known that numerous species possess the ability to hear US and echolocate in their environment. Recently, prestin has gained attention as a promising candidate for contributing to high-frequency hearing in mammals due to the following findings. (1) Prestin is a membrane protein exclusively expressed in the cochlear outer hair cells, an essential auditory cell type crucial for high-frequency hearing in animals (Zheng et al., 2000; Dallos and Fakler, 2002; Rossiter et al., 2011). (2) *In vitro* characterization of prestin has demonstrated that transmembrane voltage fluctuations can induce changes in prestin's conformation, resulting in cell contraction and elongation, a phenomenon referred to as electromobility (Ludwig et al., 2001; Bavi et al., 2021). (3) Defects in prestin have been associated with hearing loss in both humans and mice (Liberman et al., 2002; Liu, 2003; Dallos, 2008; Walton et al., 2018). These findings raise a possibility that prestin may sense US-induced transmembrane voltage fluctuations and covert to mechanical effects through its electromobility. Evolutionary studies have shown that prestin is present in nearly all mammals, and its protein sequence is highly conserved across different species (Liu et al., 2010). By comparing prestin protein sequences between echolocating and non-echolocating species, Huang et al. observed two evolutionarily significant amino acid substitutions, N7T and N308S, which are frequently found in echolocating species but not in non-echolocating ones. Intriguingly, the introduction of N7T and N308S mutants to the mouse prestin gene intensifies its sensitivity to the US (Huang et al., 2020). Accumulated studies have found that prestin forms clusters on the cell membranes (Greeson et al., 2006; Mio et al., 2008). A conformational change of prestin in response to transmembrane voltage fluctuations causes vibration in the prestin clusters, resulting in membrane deformation (Dehghani-Ghahnaviyeh et al., 2022). Interestingly, prestin (N7T, N308S) forms more clusters on the cell membrane compared to wild-type prestin (Huang et al., 2020, 2021). High-speed imaging showed that vibration of prestin (N7T, N308S)-positive cluster occurs upon US stimulation. This vibration is dependent on the electromobility of prestin and plays a crucial role in downstream calcium influx (Huang et al., 2020). These findings assume that prestin clusters may act as molecular amplifiers, lowering the threshold of US-induced bio-effects. With this capability, specific neuronal activation in the ventral tegmental area can be achieved through US stimulation (Huang et al., 2020; Wu et al., 2020). Furthermore, Fan et al. successfully stimulated dopaminergic neuron activity to induce neurotrophic expression in Parkinson's disease mice, ameliorating their motor symptoms (Fan et al., 2021). Notably, mouse prestin (N7T, N308S) only responds to 500 kHz, not other US frequencies such as 80 kHz, which are often encountered in nature. This suggests that prestin (N7T, N308S)-dependent US response involves different mechanisms compared to high-frequency hearing in auditory organs. Prestin itself is not an ion channel and, therefore, requires assistance from other endogenous components, such as mechanosensitive ion channels, to transform prestin's electromobility into ion influx and subsequent changes in membrane potential. The expression profile of downstream molecules may play a pivotal role in determining the efficiency and effects of prestin-based sonogenetics. While further evidence is necessary to elucidate the detailed mechanisms involved, these studies have demonstrated the potential of using auditory sensing proteins in the field of sonogenetics.

## Conclusion, challenges, and perspectives

Sonogenetics is an emerging approach enabling non-invasive modulation of target neurons and potential therapeutic applications. However, several issues need to be addressed to make sonogenetics more practical. First, an issue commonly encountered in sonogenetics is off-target effects. Multiple independent research teams have discovered that transcranial FUS, despite using frequencies beyond the range of hearing, can activate the peripheral auditory system as well as the motor cortex in different species (Foster and Wiederhold, 1978; Guo et al., 2018; Sato et al., 2018). To exclude indirect auditory response upon transcranial FUS stimulation, Mohammadjavadi et al. found that the smooth US rectangular waveform envelopes could directly induce motor responses without unintended peripheral auditory responses (Mohammadjavadi et al., 2019). Such parameters of FUS stimulation need to be considered in future animal studies. Second, the heterogeneous expression of US-sensing proteins in target cells makes them sensitive to US stimulation. However, many US-sensing proteins, such as various mechanosensitive ion channels, are ubiquitously present in different tissues, which raises the possibility of unexpected activation in naive cells. Orthogonality can be achieved by either utilizing sensing proteins from evolutionarily divergent species or engineering wild-type sensing proteins to boost their sensitivity to the US. For example, Huang et al. found that introducing N7T and N308S mutants to the wild-type prestin protein rendered it sensitive to the US (Huang et al., 2020). Ye et al. and Qiu et al. discovered that MscL-I92L and MscL-G22s, two prokaryotic MscL variants with higher mechanosensitivity compared to the wild-type, could sensitize neurons to FUS (Ye et al., 2018; Qiu et al., 2020). The introduction of GVs originally derived from cyanobacteria to target tissues could amplify FUS stimulation in cells bound with GVs (Hou et al., 2021). The optimized US stimulation for these engineering proteins could theoretically reduce the off-target effects in non-modified cells. Identifying more orthogonal US-sensing proteins from non-primate species or through protein engineering will enable specific modulation through FUS stimulation in clinical applications.

Although many efforts have been made to evaluate the correlation between FUS parameters and induced bio-effects (Collins and Mesce, 2022), most of them focus on only one type of tissue or cell. Tissues with different acoustic properties could exhibit distinct bio-effects upon FUS stimulation. In fact, FUS stimulation with almost the same parameters can induce opposite neuronal activities (Wang et al., 2020). The key factors determining the cellular sensitivity to the US and the resulting bio-effects need to be comprehensively explored. For example, factors such as cell stiffness (Bergman et al., 2021) and cytoskeleton composition (Noriega et al., 2013; Duque et al., 2022) are important for US sensitivity. The correlations among cells with different acoustic properties and FUS stimulation need to be comprehensively studied to better understand the mechanisms of sonogenetics. Besides the complexity of biocomponents, dependent on the design of transducers, various types of US wave propagation can be triggered, potentially resulting in distinct bioeffects on cells (Lo et al., 2021; Chu et al., 2022). Standardizing transducer designs and conducting comparative studies on different transducers are crucial for condition optimization in different physiological situations.

One advantage of optogenetics is multiplexing, offering the capability to use different light wavelengths to stimulate corresponding light-sensitive sensors and their related signaling pathways. For example, channelrhodopsin-2 and halorhodopsin, two photosensitive cation and anion channels, can be specifically activated by 470 and 589 nm light, respectively (Pastrana, 2011). Therefore, optogenetics can depolarize and hyperpolarize target neurons as needed. In addition to ion specificity, the identification of various photosensitive ion channels with different gating and opening durations allows for transient and sustained effects through pulsed light (Yizhar et al., 2011). Multiplexing in optogenetics provides flexibility in experimental design as well as clinical applications. Sonogenetics also has the potential for multiplexing, as a few studies have shown that different parameters of FUS can modulate distinct responses of US-sensing proteins. For instance, it has been demonstrated that GVs can be vibrated and collapsed by low-intensity and high-intensity US, respectively, opening up various applications (Bar-Zion et al., 2021; Wu et al., 2023). Huang et al. further showed that prestin (N7T, N308S) can be efficiently activated by 0.5 MHz FUS but not by other tested frequencies (Huang et al., 2020). The discovery of more US-sensitive proteins responsive to specific US parameters will greatly extend the toolkits of sonogenetics.

## Author contributions

H-CW: Writing – original draft. T-NP: Writing – original draft. C-LK: Writing – original draft. C-KY: Writing – review & editing. Y-CL: Writing – original draft, Writing – review & editing.
